# Two featured series of rRNA-derived RNA fragments (rRFs) constitute a novel class of small RNAs

**DOI:** 10.1371/journal.pone.0176458

**Published:** 2017-04-25

**Authors:** Ze Chen, Yu Sun, Xiaojun Yang, Zhenfeng Wu, Kaifei Guo, Xiaoran Niu, Qingsong Wang, Jishou Ruan, Wenjun Bu, Shan Gao

**Affiliations:** 1 State Key Laboratory of Veterinary Etiological Biology, Lanzhou Veterinary Research Institute, Chinese Academy of Agricultural Science, Lanzhou, Gansu, China; 2 College of Life Sciences, Nankai University, Tianjin, Tianjin, China; 3 Faculty of Ecotourism, Southwest Forestry University, Kunming, Yunnan, China; 4 School of Mathematical Sciences, Nankai University, Tianjin, China; 5 Tianjin Research Center of Basic Medical Sciences, Tianjin Medical University, Tianjin, China; 6 Institute of Statistics, Nankai University, Tianjin, China; Huazhong University of Science and Technology, CHINA

## Abstract

In this study, we reported two featured series of rRNA-derived RNA fragments (rRFs) from the small RNA sequencing (sRNA-seq) data of *Amblyomma testudinarium* using the Illunima platform. Two series of rRFs (rRF5 and rRF3) were precisely aligned to the 5' and 3' ends of the 5.8S and 28S rRNA gene. The rRF5 and rRF3 series were significantly more highly expressed than the rRFs located in the body of the rRNA genes. These series contained perfectly aligned reads, the lengths of which varied progressively with 1-bp differences. The rRF5 and rRF3 series in the same expression pattern exist ubiquitously from ticks to human. The cellular experiments showed the RNAi knockdown of one 20-nt rRF3 induced the cell apoptosis and inhibited the cell proliferation. In addition, the RNAi knockdown resulted in a significant decrease of H1299 cells in the G2 phase of the cell cycle. These results indicated the rRF5 and rRF3 series were not random intermediates or products during rRNA degradation, but could constitute a new class of small RNAs that deserves further investigation.

## Introduction

Small RNAs (sRNAs) are usually non-coding RNA molecules, typically not more than 200 nt in length. The well-known classes of sRNAs include microRNAs (miRNAs) [1], small interfering RNAs (siRNAs) [2], piwi-interacting RNA (piRNAs) [3], transcription initiation RNA (tiRNAs) [4], small nuclear RNA (snRNAs) [5], small nucleolar RNA (snoRNAs) [6] and *etc*. Following the discovery of siRNAs, trans-acting siRNAs (tasiRNAs) [7] and natural antisense transcripts siRNAs (natsiRNAs) [8] have been identified as two types of siRNAs in plants. With the large-scale use of small RNA sequencing (sRNA-seq) based on the Next Generation Sequencing (NGS) technologies, many novel classes of sRNAs have been identified to enrich our knowledge. For example, a novel class of sRNAs named tRNA-derived RNA fragments (tRFs) was introduced using the sRNA-seq data of the human prostate cancer cell line by 454 deep sequencing in the year of 2009 [9]. According to this study, the tRFs were measured to be the most abundant class of sRNAs, second only to miRNAs. Three series of tRFs (tRF-5, tRF-3 and tRF-1) have been identified and characterized to indicate they are not random degradation intermediates. Particularly, tRF-1 has been proved to be required for cell proliferation by the MTT assays.

Compared to mRNAs(~5%) and tRNAs(~15%), rRNAs (~80%) are the most abundant RNA in the total RNA of mammalian cells [10]. The rRNA-derived RNA fragments (rRFs) are usually removed as the contamination from random degradation in the RNA-seq or sRNA-seq data analysis [11]. In recent years, a few scientists are attempting to investigate some specific rRFs with their biological functions. One previous study discovered a new type of siRNAs, known as QDE-2-interacting small RNAs (qiRNAs) induced by DNA damage were mainly originated from rDNA loci [12]. Another study demonstrated that various new small noncoding RNAs (ncRNAs) including piRNA-like 5-half-tRNAs and 28S rRFs co-immunoprecipitated with tRNase ZL in human kidney 293 cells and could work as small guide RNAs (sgRNAs) for tRNase ZL *in vivo* as well as *in vitro* [13]. It was also reported that DNA double-strand breaks (DSBs) may be introduced within the rDNA repeats, which could then trigger the production of rDNA-specific sRNAs that mediate DSB repair on damaged repetitive rDNAs [14].

Due to the flaws in the library construction protocols or the sequencing length and depth, the previous studies could not provide adequate information to systematically investigate the rRFs. In this study, we constructed the small RNA library of hard ticks (*Amblyomma testudinarium* Koch, 1844) using the newest protocol and sequenced this library on the Illumina Platform. As a result, we reported two featured series of rRFs (rRF5 and rRF3). The profiling and characterization of the rRF5 and rRF3 series indicated that they were not random intermediates or products during rRNA degradation, but could constitute a novel class of sRNAs that deserves further investigation. The small RNA-seq data is available at the NCBI SRA database under the project accession number SRP084097.

## Results

**Fig 1:**
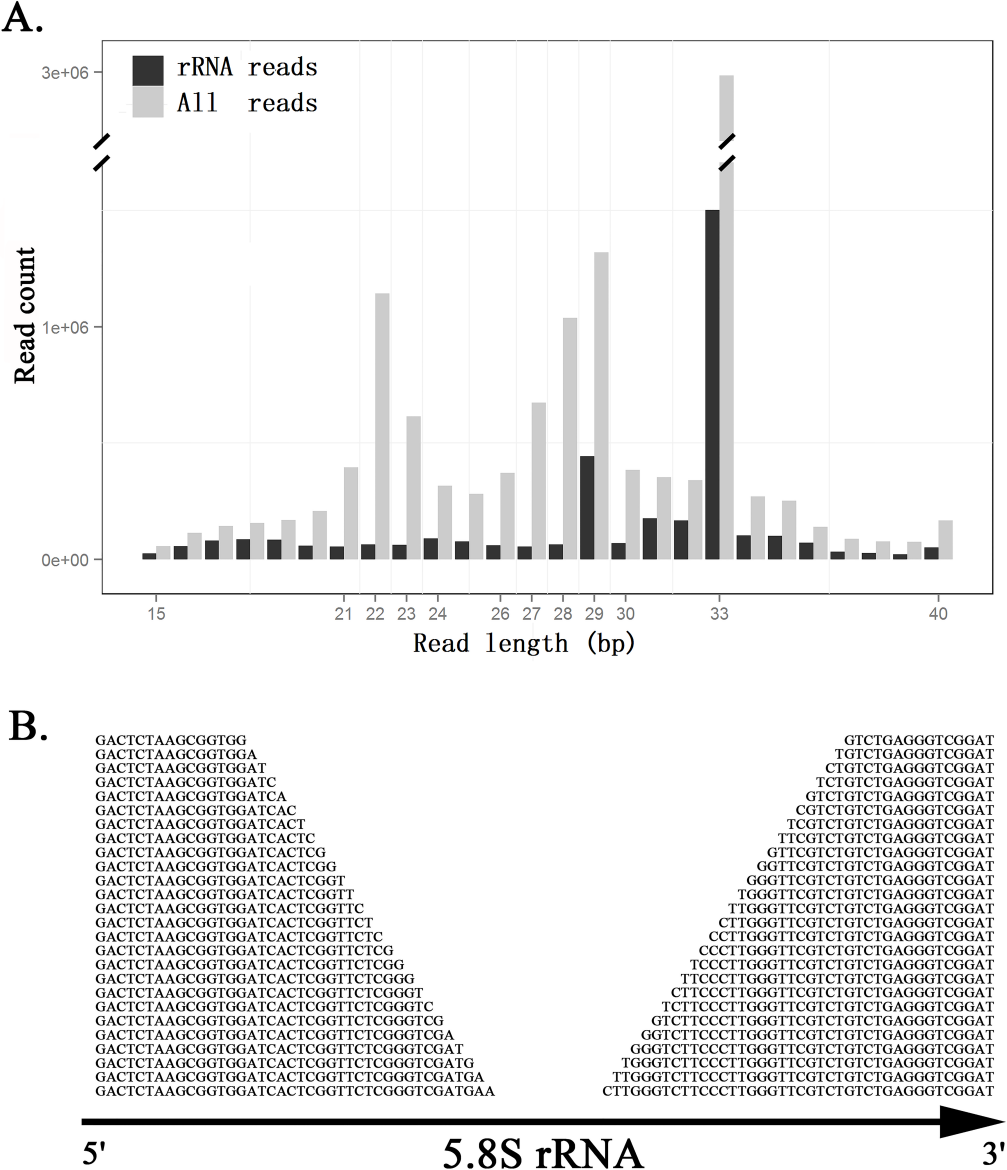
Length distribution of the small RNA-seq data. This small RNA sequencing (sRNA-seq) data was from two adult female ticks (*Amblyomma testudinarium* Koch, 1844) using the single-end (PE) 50-bp sequencing on the Illunima platform. After data cleaning and quality control, a total of 13,723,268 raw reads were processed to 12,134,881 cleaned reads for statistics calculation. (A). All reads and rRNA reads represent all the cleaned reads with lengths above 15 bp and the reads aligned to the reference rDNA sequence (Genbank: AF291874.1) allowing one gap or mismatch, respectively. (B). The lengths of rRF5 and rRF3 series from the 5.8S and 28S gene vary progressively with 1-bp differences. This pattern is conserved in ticks and human.

Total RNA was extracted from two adult female ticks (*Amblyomma testudinarium* Koch, 1844) and pooled together to construct one sRNA library, which was sequenced on the Illumina Hiseq 2500 platform. After data cleaning and quality control, a total of 13,723,268 single-end 50-bp raw reads (0.69 Gbp data) were processed to 12,134,881 cleaned reads, with the Q20 percentage of 99.3%. The length distribution of total cleaned reads (**Fig 1A**) ranged from 15 bp to 40 bp and was enriched in two expected regions, which were the miRNA and siRNA region (21~24 bp) and the piRNA region (26~30 bp). The two highest histogram bins at the 29-bp and 33-bp position could contain a large number of PCR-duplicated reads, which needs to be analyzed in the following study. Using sequence alignment, 30.4% (3,688,594/12,134,881) of the cleaned reads could be mapped to the reference rDNA sequence of *Amblyomma americanum* (Genbank: AF291874.1). The length distribution of these rRNA reads (**Fig 1A**) suggested the lengths of rRFs could span a wide range. This high mapping rate of 30.4% and the wide length range of rRFs were out of expectation, suggesting the rRF abundance had been underestimated in the previous studies [9].

**Fig 2:**
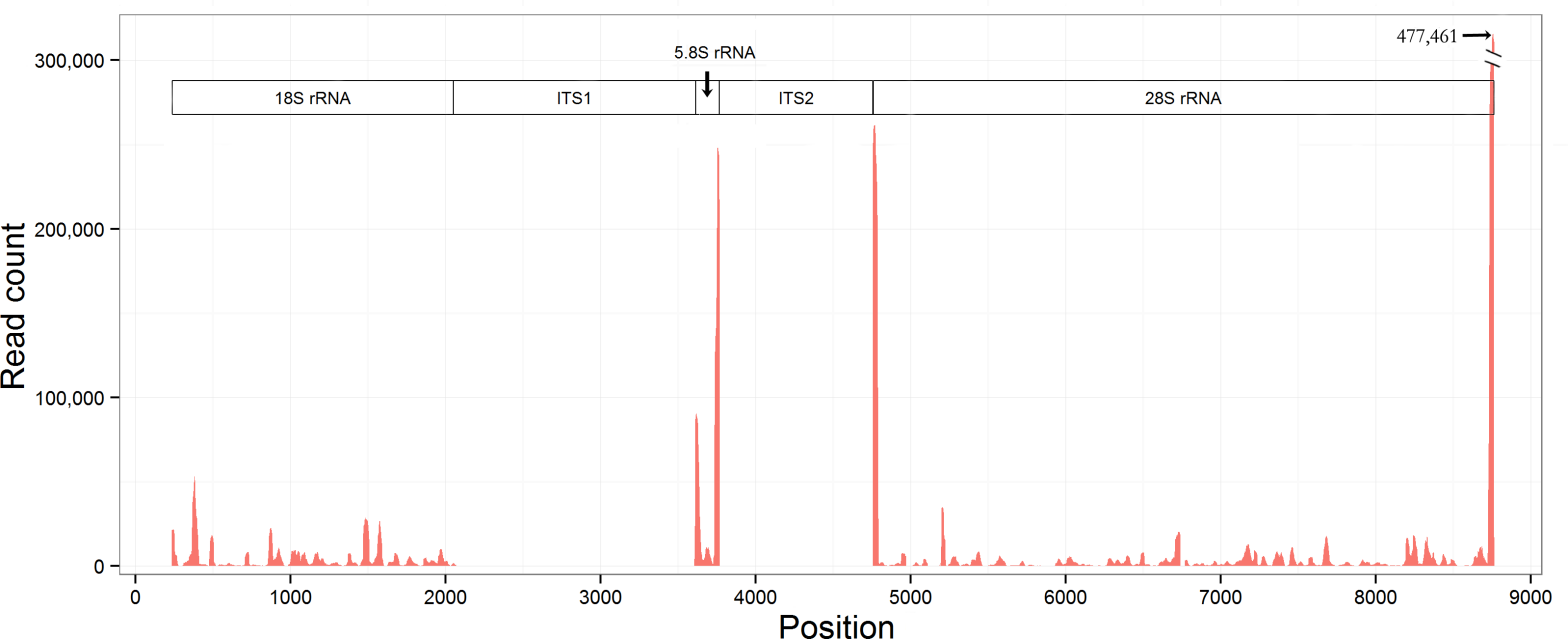
Distribution of aligned reads on the reference rDNA sequence. This figure shows the count distribution of all aligned reads on the reference rDNA sequence (Genbank: AF291874.1). This rDNA sequence is from the sense strand of DNA. ITS1 and ITS2 represents the internal transcribed spacer 1 and the internal transcribed spacer 2. Two extremely abundant sRNAs (TATTGAGGCTTAGCCTCTGACTGGAAGGTTTGT and GAGGCTTAGCCTCTGACTGGAAGGTTTGT) were not used to plot this figure to avoid the bias brought by the possible PCR duplication.

The alignment result showed more than 95% rRFs could be derived from the 18S, 5.8S and 28S rRNA gene, while only a few of rRFs could be derived from the internal transcribed spacer 1 (ITS1) and the internal transcribed spacer 2 (ITS2) (**Fig 2**). Among the aligned reads, 99.98% (3,687,679/3,688,594) were on the sense strand. No double-stranded rRFs were found to support the finding in the previous study[14]. The alignment result also showed rRFs were significantly enriched on the 5' and 3' ends of the 5.8S and 28S rRNA gene, but not enriched on the 5' and 3' ends of the 18S rRNA gene (**Fig 2**). Following the nomenclature used for tRFs [9], we defined two series of rRFs (rRF5 and rRF3) that were precisely aligned to the 5' and 3' ends of the 5.8S and 28S rRNA gene (**S1 File**). Generally speaking, the rRF5 and rRF3 series were significantly more highly expressed than the rRFs located in the body of the rRNA genes. The rRF3 series were significantly more highly expressed than the rRF5 series. In addition, we found a new pattern that the lengths of rRF5 and rRF3 series varied progressively with 1-bp differences (**Fig 1B**). This pattern suggested the rRF5 and rRF3 series were not random intermediates or products during rRNA degradation.

**Fig 3:**
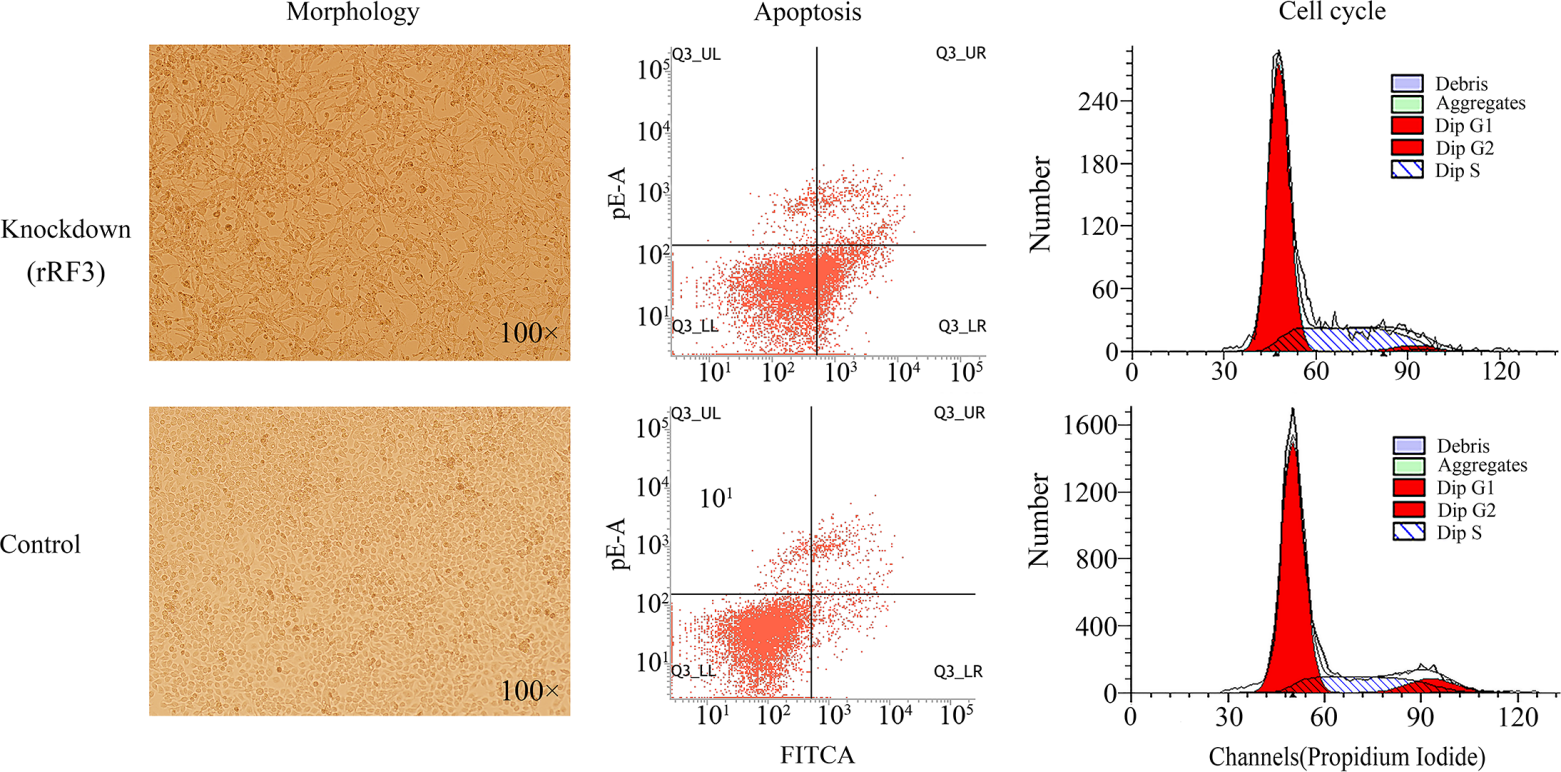
RNAi knockdown of rRF3 in H1299 cells. One 20-nt rRF3 "ATTCGTAGACGACCTGCTTC" and its control "CGTACGCGGAATACTTCGA" were selected to use as target sequences for the pSIREN-RetroQ vector construction. The cell morphology was observed using a CKX71 inverted microscopy (Olympus, Japan). The proliferation percentage of rRF3 knockdown cells and control cells is 24.02% and 4.17%, respectively. The cell cycle percentages of rRF3 knockdown cells include 68.8%, 2.72% and 28.48% for the G1, G2 and S phase. The cell cycle percentages of control cells include 71.2.8%, 7.93% and 20.87% for the G1, G2 and S phase.

The further analysis showed the two highest histogram bins at the 29-bp and 33-bp position (**Fig 1A**) could be attributed to several sRNAs with their variants. The highest abundant 33-bp and 29-bp sRNA was "TATTGAGGCTTAGCCTCTGACTGGAAGGTTTGT" and "GAGGCTTAGCCTCTGACTGGAAGGTTTGT" belonging to the rRF3 series. Their read count reached 11.73% (1,423,580/2,134,881) and 3.22% (390,952/2,134,881) of the total cleaned reads, respectively, without considering their variants. These two sRNAs were not used to plot Fig 3 to avoid the bias brought by the possible PCR duplication. Another abundant 33-bp sRNA "TCCGGCGTGGTCTAGTGGCTAGGATATCTGGCT" comprised 2.37% (288,062/12,134,881) of the total cleaned reads. This sRNA could not be aligned to the reference rDNA sequence.

Since rRNAs are the most abundant RNA and play a very important role in cellular functions, the rRF5 and rRF3 series could exist ubiquitously from ticks to human. To validate this hypothesis, we aligned the public small RNA-seq data (**Materials and methods**) to the human 45S pre-rRNA sequence (RefSeq: NR_046235.1). The results validated our hypothesis that the rRF5 and rRF3 series in human have the same enrichment levels on the 5' and 3' ends of the 5.8S and 28S rRNA gene as the rRF5 and rRF3 series in ticks (**S1 File**). Particularly, the lengths of rRF5 and rRF3 series also varied progressively with 1-bp differences in human. The cross-species conservation of the rRF5 and rRF3 series generated our interest to investigate their biological functions. From the human 28S rRF3 series, one 20-nt rRF3 "ATTCGTAGACGACCTGCTTC" named as hsa-rRF3-000001 was selected to design a short hairpin RNA (shRNA), which was used to conduct RNA interference (RNAi) experiments for the knockdown of hsa-rRF3-000001 (**Materials and methods**). The RNAi knockdown of hsa-rRF3-000001 was conducted by the transfection of the shRNA-containing pSIREN-RetroQ vector into the H1299 cells. The transfected cells were tested to measure the changes in their proliferation, apoptosis and cell cycle (**S2 File**). The experimental results (**Fig 3**) showed the RNAi knockdown of hsa-rRF3-000001 in the H1299 cells significantly induced the cell apoptosis to 5.76 times (P<0.01) and inhibited the cell proliferation to 72.73% (P<0.01). In addition, the RNAi knockdown of hsa-rRF3-000001 resulted in a significant decrease of H1299 cells in the G2 phase of the cell cycle. These results suggest the rRF5 and rRF3 series could constitute a novel class of sRNAs.

## Discussion

In this study, we reported two featured series of rRNA-derived RNA fragments (rRFs) from the small RNA sequencing (sRNA-seq) data of two hard ticks using the Illunima platform. To ensure the accuracy of the sRNA-seq data, we applied a new protocol to construct sRNA library using total RNA without size selection. This new protocol plus the high sequencing depth (13,723,268 reads in total) were used to perverse the original sRNA distribution to the utmost extent. With the help of these improvements, we identified two series of rRFs (rRF5 and rRF3), which could constitute a novel class of sRNAs. The rRF5 and rRF3 series in the same expression pattern exist ubiquitously in animals. Although the mechanism of the rRF5 and rRF3 biogenesis is not clear yet, the RNAi experiments preliminarily proved the cellular functions of one member of the rRF5 and rRF3 series in the H1299 cells. Future Work should be made to investigate what regulates the rRF5 and rRF3 series and if they can regulate the rRNA metabolism. Another phenomenon which deserves further study is the rRF5 and rRF3 series from the 5.8S and 28S rRNA gene were found significantly more highly expressed than those from the 18S rRNA gene. The ribosome contains two subunits, the large subunit (LSU) and small subunit (SSU). In animals, 5.8S and 28S rRNAs belong to LSU and 18S rRNAs belong to SSU. The human 18S, 5.8S and 28S mature rRNAs are derived from the 47S pre-rRNAs. The 18S rRNAs have a different biogenesis pathway from the 5.8S and 28S rRNAs. This suggests these rRNAs could have different regulation mechanisms.

## Materials and methods

### Ethics statement

This research was carried out according to the Guidelines for the Use of Animals of the People's Republic of China. The ticks were captured from buffalo with the farm owner’s permission. In addition, there was no endangered or protected species involved in the research.

### Sample preparation, sRNA library construction and sequencing

Two adult female ticks (*Amblyomma testudinarium* Koch, 1844) were captured from buffalo in Xishuangbanna, Yunnan province of China (22°N, 101°E) on 9^th^ April 2016. Two living ticks were immediately frozen with liquid nitrogen at -196°C and preserved at -80°C for 10 days before the RNA extraction using Trizol Reagent (Thermo Fisher Scientific, USA). After that, 3 μg RNA was used to construct one small RNA library using NEBNext Multiplex Small RNA Library Prep Set for Illumina (NEB, USA) following the manufacturer’s instructions. Briefly, 3' single-stranded adapters were directly and specifically ligated to 3' ends of the total RNA without size selection. After the 3' ligation reaction, RT Primers were hybridized to the ligated RNA and the excess of 3' adapters, which transformed the single-stranded DNA adapters into double-stranded DNA molecules. This step is important to prevent adaptor-dimer formation, besides, dsDNAs are not substrates for ligation mediated by T4 RNA Ligase 1 and therefore cannot ligate to 5´single-stranded adapters in the subsequent step. Then, 5´adapters were ligated to 5´ends of the total RNA. Only the first-strand cDNA was synthesized using MMLV Reverse Transcriptase with the reduced RNase H activity. PCR amplification of the first-strand cDNA was performed using LongAmp Taq 2X Master Mix and PCR Primers with the index "AGTGGACA" for Illumina sequencers. PCR products were purified using polyacrylamide gel (8%) electrophoresis (100V, 80 min). DNA fragments with sizes estimated as 130~160 bp in the bright gel band were recovered and dissolved in 8 μL elution buffer. The sRNA library quality was assessed on the Bioanalyzer 2100 system (Agilent, USA) using DNA High Sensitivity Chips. The clustering of the index-coded samples was performed on the cBot Cluster Generation System using TruSeq SR Cluster Kit v3-cBot-HS (Illumina, USA) according to the manufacturer’s instructions. After cluster generation, the sRNA library was sequenced using the single-end (PE) 50-bp sequencing on the Illumina Hiseq 2500 platform.

### Data analysis

The software Fastq_clean was used for sRNA-seq data cleaning and quality control [15]. Fastq_clean is a Perl based pipeline to clean DNA-seq [16], RNA-seq [17] and sRNA-seq data [11] with quality control and can be downloaded from the website (https://github.com/gaoshanT/Fastq_clean). The software BWA included in Fastq_clean was used to align the cleaned sRNA data to the reference rDNA sequence (Genbank: AF291874.1) of *Amblyomma americanum* and the reference rRNA sequence (RefSeq: NR_046235.1) of human, allowing one mismatch or gap and filtering reads shorter than 15 bp. The alignment results were observed and double-checked using the software Tablet v1.15.09.01. Statistics and plotting were conducted using the software R v2.15.3 with the Bioconductor packages [18]. The public small RNA-seq dataset used to identify the rRF5 and rRF3 series in human was downloaded from the NCBI SRA database under the project accession number SRP002272 (**S2 File**).

### RNAi and cellular experiments

Based on the shRNA design protocol (**S2 File**), one 20-nt rRF3 "ATTCGTAGACGACCTGCTTC" and its control "CGTACGCGGAATACTTCGA" were selected to use as target sequences for the pSIREN-RetroQ vector construction (Clontech, USA). The BLAST results confirmed that the selected sequences were not homologous to other human gene sequences. At 24 h prior to transfection, the H1299 cells were trypsinized (0.25% trypsin in DMEM) and seeded in 6-well plates at a density of 2 x 10^5^ cells/well (sample). The cells were cultured for 24 h and reached 80% confluence, which was confirmed by a CKX71 inverted microscopy (Olympus, Japan). The transfection of 2 μg vectors into each well (sample) was performed using Lipfectamine 2000 (Life technology, USA) following the manufacturer's instructions.

The cellular experiments were performed in two groups, the rRF3 knockdown group (three samples) and the control group (three samples) to measure the cell proliferation, apoptosis and cell cycle at 96 h after transfection. Cell proliferation was measured using Vybrant MTT Cell Proliferation Assay Kit (ThermoFisher Scientific, USA) and cell apoptosis was measured using FITC Annexin V Apoptosis Detection Kit I (BD Biosciences, USA). To measure the cell cycle, the harvested cells in each sample were fixed in 75% (v/v) ethanol and preserved at -20°C overnight. Then, these cells were washed with PBS and resuspended in propidium iodide (PI) staining buffer (PBS with 50 μg/ml PI and 100 μg/ml DNase-free RNase) at 4°C for 30 min. All the samples for the detection of cell apoptosis and cell cycle were finally analyzed using a FACSCalibur flow cytometer (BD Biosciences, USA).

## Supporting information

S1 File The rRF5 and rRF3 series. The results validated our hypothesis that the rRF5 and rRF3 series in human have the same enrichment levels on the 5' and 3' ends of the 5.8S and 28S rRNA gene as the rRF5 and rRF3 series in ticks.
(XLSX)

S2 File Additional analysis and experiments. Additional analysis in human by using public data were conducted to validate our findings. Additional experiments were also conducted to validate our findings.
(DOC)
