# Supplementary material for: Two featured series of rRNA-derived RNA fragments (rRFs) constitute a novel class of small RNAs
Source: PLoS One. 2017 Apr 25;12(4):e0176458. doi: 10.1371/journal.pone.0176458 (PMC5404876; doi:10.1371/journal.pone.0176458)
Supplement: S2 File — Additional analysis in human by using public data were conducted to validate our findings. Additional experiments were also conducted to validate our findings. (DOC) [file pone.0176458.s002.doc]

# Supplementary 2

### **2.1 The rRF5 and rRF3 series in human**

The public small RNA-seq dataset used to identify the rRF5 and rRF3 series in human was downloaded from the NCBI SRA database under the project accession number SRP002272. This dataset included 15 clinical samples, which are three normal liver tissues, one HBV-infected liver tissue, one severe chronic Hepatitis B liver tissue, two Hepatitis B virus (HBV) positive Hepatocellular Carcinoma (HCC) tissues, one Hepatitis C virus (HCV) positive HCC tissue, one HCC tissue without HBV or HCV and six controls. The data analysis (e.g. alignment) was conducted following the procedure used in our previous study [1]. The analysis of this dataset demonstrated the rRF5 and rRF3 series in human have the same pattern as the rRF5 and rRF3 series in ticks. Here is the results of the data SRR039612.


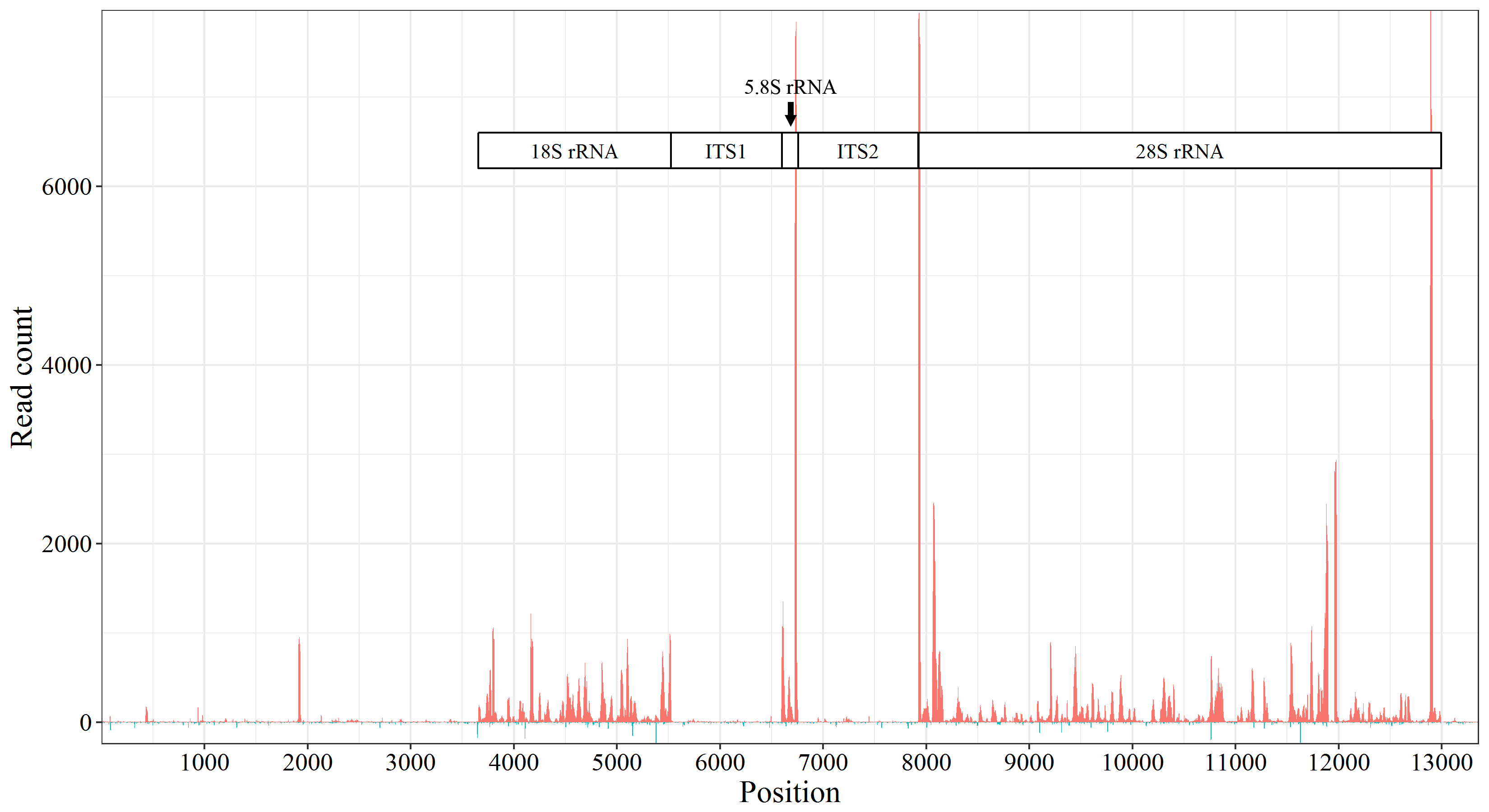


**Figure S1. Distribution of aligned reads on the 45S pre-rRNA sequence.** After data cleaning and quality control, a total of 15,656,640 raw reads were processed to 15,594,578 cleaned reads for statistics calculation. This figure shows the count distribution of all aligned reads on the reference 45S pre-rRNA (RefSeq: NR_046235.1). This sequence is from the sense strand of DNA. ITS1 and ITS2 represents the internal transcribed spacer 1 and the internal transcribed spacer 2.

### **2.2 The pSIREN-RetroQ vector and shRNA design**


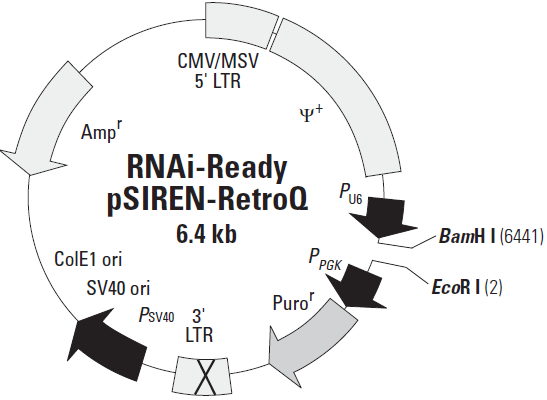


**Figure S2. Restriction map and cloning site of the pSIREN-RetroQ vector.** Unique restriction sites are in bold. The pSIREN-RetroQ is provided as a linearized vector digested with BamH I and EcoR I. The shRNA should be inserted into between the BamH I and EcoR I site one the vector.





**Figure S3. From plasmid to siRNA duplex.** This target sequence is cloned downstream of a Pol III promoter in an expression vector for gene silencing in mammalian cells. A hairpin loop sequence is located between the sense and antisense sequences on each complementary strand.

### **2.3 The results of cellular experiments in details**

**Table S1. The results of cellular experiments**

|  | **rRF3 knockdown** | | | **Control** | | | **Ratio** | **P-value** |
| --- | --- | --- | --- | --- | --- | --- | --- | --- |
| **Apoptosis(%)** | 24.02 | 23.76 | 24.13 | 4.17 | 4.23 | 4.08 | 5.762 | 2.4E-06 |
| **Cycle G1(%)** | 68.8 | 67.53 | 69.17 | 71.2 | 70.8 | 72.5 | 0.958 | 0.01372 |
| **Cycle G2(%)** | 2.72 | 2.68 | 2.83 | 7.93 | 8.01 | 7.89 | 0.345 | 1.8E-07 |
| **Cycle S(%)** | 28.48 | 29.79 | 28 | 20.87 | 21.19 | 19.61 | 1.399 | 0.00036 |
| **Proliferation** | 0.52 | 0.44 | 0.47 | 0.37 | 0.31 | 0.36 | 1.375 | 0.01344 |

The cellular experiments were performed in two groups, the rRF3 knockdown group (three samples) and the control group (three samples) to measure the cell proliferation, apoptosis and cell cycle at 72 h after transfection. Ratio was equal to the average of three control samples divided by the average of three rRF3 knockdown samples. P-value was estimated using the t-test function in the excel software.

**Table S2. The results of MTT**

|  | **1** | **2** | **3** | **4** | **Mean** |
| --- | --- | --- | --- | --- | --- |
| **Knockdown 1** | 0.402 | 0.324 | 0.329 | 0.425 | 0.37 |
| **Knockdown 2** | 0.339 | 0.404 | 0.249 | 0.253 | 0.3113 |
| **Knockdown 3** | 0.387 | 0.345 | 0.302 | 0.403 | 0.3593 |
| **Control 1** | 0.548 | 0.484 | 0.45 | 0.586 | 0.517 |
| **Control 2** | 0.525 | 0.354 | 0.403 | 0.46 | 0.4355 |
| **Control 3** | 0.537 | 0.41 | 0.422 | 0.517 | 0.4715 |

The MTT experiments were performed in two groups, the rRF3 knockdown group (three samples) and the control group (three samples). For each sample, the data from four wells were collected for statistics.
